# Design, Synthesis, In Vitro Biological Evaluation and In Silico Molecular Docking Study of Benzimidazole-Based Oxazole Analogues: A Promising Acetylcholinesterase and Butyrylcholinesterase Inhibitors

**DOI:** 10.3390/molecules28207015

**Published:** 2023-10-10

**Authors:** Rafaqat Hussain, Fazal Rahim, Hayat Ullah, Shoaib Khan, Maliha Sarfraz, Rashid Iqbal, Faiza Suleman, Mohammad Khalid Al-Sadoon

**Affiliations:** 1 Department of Chemistry, Hazara University, Mansehra 21120, Pakistan; rafaqathussain0347@gmail.com; 2 Department of Chemistry, University of Okara, Okara 56130, Pakistan; 3 Department of Chemistry, Abbottabad University of Science and Technology (AUST), Abbottabad 22500, Pakistan; shoaibkhanswati@gmail.com; 4 Department of Zoology, Wildlife and Fisheries, University of Agriculture Faisalabad, Sub Campus Toba Tek Singh, Faisalabad 36050, Pakistan; maliha.sarfraz@yahoo.com; 5 Department of Agroecology-Climate and Water, Aarhus University, Blichers Allé 20, 8830 Tjele, Denmark; rashid.iqbal@iub.edu.pk; 6 Department of Agronomy, Faculty of Agriculture and Environment, The Islamia University of Bahawalpur, Bahawalpur 63100, Pakistan; 7 Department of Botany, Government College University, Lahore 54000, Pakistan; 8 Department of Zoology, College of Science, King Saud University, P.O. Box 2455, Riyadh 11451, Saudi Arabia; msadoon@ksu.edu.sa

**Keywords:** synthesis, benzimidazole, oxazole, AChE and BuChE, molecular docking study, structure-activity relationship

## Abstract

Alzheimer’s disease (AD) is a degenerative neurological condition that severely affects the elderly and is clinically recognised by a decrease in cognition and memory. The treatment of this disease has drawn considerable attention and sparked increased interest among the researchers in this field as a result of a number of factors, including an increase in the population of patients over time, a significant decline in patient quality of life, and the high cost of treatment and care. The current work was carried out for the synthesis of benzimidazole-oxazole hybrid derivatives as efficient Alzheimer’s inhibitors and as a springboard for investigating novel anti-chemical Alzheimer’s prototypes. The inhibition profiles of each synthesised analogue against acetylcholinesterase (AChE) and butyrylcholinesterase (BuChE) enzymes were assessed. All the synthesized benzimidazole-based oxazole analogues displayed a diverse spectrum of inhibitory potentials against targeted AChE and BuChE enzymes when compared to the reference drug donepezil (IC_50_ = 2.16 ± 0.12 M and 4.50 ± 0.11 µM, respectively). The most active AChE and BuChE analogues were discovered to be analogues 9 and 14, with IC_50_ values of 0.10 ± 0.050 and 0.20 ± 0.050 µM (against AChE) and 0.20 ± 0.050 and 0.30 ± 0.050 µM (against BuChE), respectively. The nature, number, position, and electron-donating and -withdrawing effects on the phenyl ring were taken into consideration when analysing the structure-activity relationship (SAR). Molecular docking studies were also carried out on the active analogues to find out how amino acids bind to the active sites of the AChE and BuChE enzymes that were being studied.

## 1. Introduction

**Figure 1:**
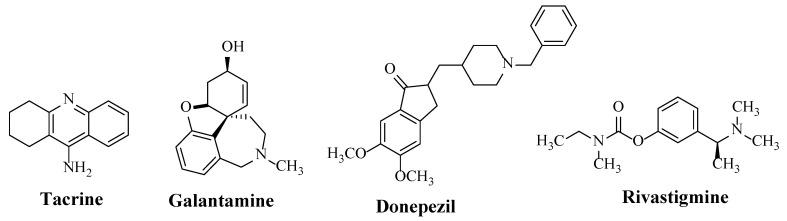
Available drug of AD with AChE inhibitory activity.

Alzheimer’s disease is mainly caused by cholinesterase (AChE and BuChE) enzymes. These enzymes primarily work to hydrolyze acetylcholine into choline acetic acid [1]. These result in a lack of acetylcholine in the hippocampus and cortex of the brain, which are linked to important psychological activities [2] and result in the development of Alzheimer’s disease (AD), a persistent and irreversible brain illness. The cholinergic system of the brain continues to be affected, which frequently results in confusion, memory loss, cognitive impairment, difficulty solving problems, and thinking difficulties [3,4,5]. Additionally, because these enzymes are in charge of the neurotoxic β-amyloid accumulation that results in neuronal cell apoptosis, AD is the main cause of dementia in an ageing society. The best course of action for treating AD is to target both of these enzymes [6,7] by concentrating on the acetylcholinesterase enzyme, which has two binding sites: a peripheral location for the interaction of β-amyloid and a catalytic site for the hydrolysis of acetylcholine. In the form of the AChE-Aβ complex, it induces neurotoxicity as a result of its interaction with proteins. Furthermore, BuChE is mostly found in the heart, lungs, kidneys, liver, gut, and serum, whereas AChE is found in the cholinergic brain, neurons, and muscle [8,9]. The primary role of these enzymes is to break down substances that contain esters; AChE is predominant in the brain, and as acetylcholine activity gradually declines, BuChE function increases. To block the potential of these enzymes, a powerful medication is still required [10]. The FDA has approved a number of medications, including donepezil, rivastigmine, tacrine, and galantamine, for the treatment of Alzheimer’s. Tacrine and Rivastigmine have been used to inhibit both AChE and BuChE, while Donepezil and Galantamine have been used to inhibit AChE [11] (Figure 1).

**Figure 2:**
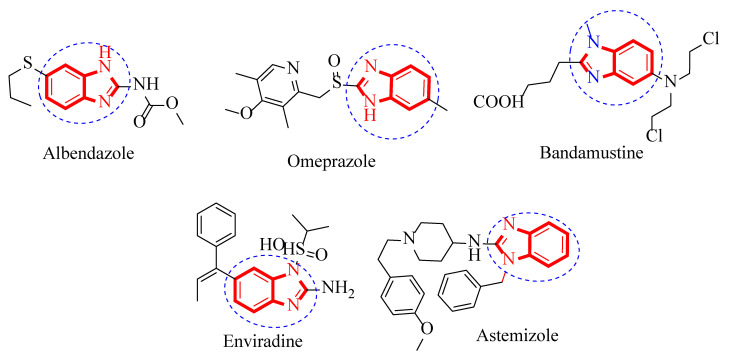
Drug comprising benzimidazole moiety.

Antioxidants [12], anticancer agents [13], CNS stimulants [14], antiviral agents [15], anticoagulants [16], anti-inflammatory agents [17], anti-parasitic agents [18], anti-microbial agents [19], and blood pressure lowering agents [20] have all been found in benzimidazole and its derivatives. There are numerous bioactive drugs used in the market that contain benzimidazole skeletons in their structures, such as bandamustine, enviradine, astemizole, albendazole, and omeprazole (Figure 2) [21].

**Figure 3:**
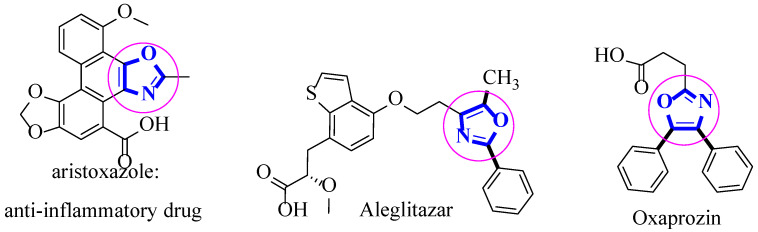
Bioactive drugs containing oxazole skeleton.

Oxazole scaffolds play a vital role in the design and development of several biologically active pharmaceutical drugs with interesting biological profiles, natural products, functional materials, and ligand frameworks [22]. Oxaprozin, aleglitazar, and aristoxazole (Figure 3) are examples of naturally occurring compounds that contain oxazole, which is a physiologically active scaffold [23,24].

**Figure 4:**
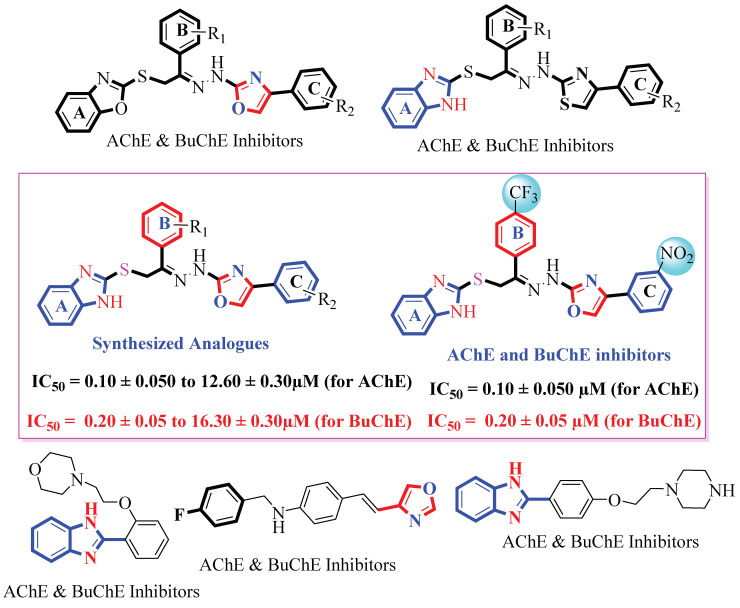
Rational of the current study.

We had already synthesized several N-containing heterocyclic compounds as potent inhibitors of alpha-amylase, alpha-glucosidase, urease, β-glucuronidase, thymidine phosphorylase, AChE and BuChE enzymes [25,26,27]. Furthermore, the molecular hybridization approach has been widely employed for the design and synthesis of hybrid analogues for the treatment of Alzheimer’s disease. This approach mainly involves the combining of two or more than two different pharmacophore moieties in a single molecule having a common skeleton. These hybrid molecules may have advantages over standard drugs. In the current work, we used molecular hybridization to combine the biologically important two heterocyclic moieties; benzimidazole and oxazole, to obtain new hybrid molecules (Figure 4). As earlier discussed, both benzimidazole and oxazole moieties are very important for the treatment of Alzheimer’s disease, and thus hybrid analogues containing benzimidazole and oxazole moieties were synthesized and evaluated for their in vitro AChE and BuChE and molecular docking studies thereafter. Keeping in mind the biological importance of benzimidazole [28,29,30] and oxazole [31] scaffolds (Figure 4), in this study, we designed and synthesized hybrid analogues based on benzimidazole-bearing oxazole derivatives to further explore the AChE and BuChE inhibition profiles for the better treatment of Alzheimer’s disease.

## 2. Results and Discussion

### 2.1. Chemistry

**Scheme 1:**
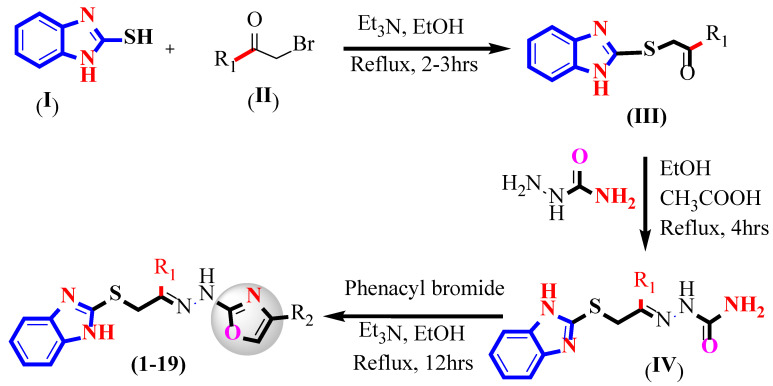
Synthesis of benzimidazole-oxazole hybrid derivatives (**1**–**19**).

The synthesis of hybrid benzimidazole-based oxazole derivatives was completed in three steps:

First, the reaction mixture of 2-marcaptobenzimidazole (I, 1 mmol) and 2-bromoacetophenone (II, 1 mmol) was mixed and refluxed for 2–3 h in ethanol (10 mL) in the presence of triethylamine as a catalyst to produce the first intermediate product (III) [32]. The second intermediate product (IV) was produced by treating the intermediate (III) and semicarbazide in equal amounts in ethanol (10 mL) and glacial acetic acid as catalysts and refluxing the mixture for around 4 h. After the reaction was complete, the mixture was cooled to room temperature, and the precipitate solid that was produced was filtered and washed with *n*-hexane. TLC plates had been used to keep tabs on the reaction’s development. An equivalent quantity of intermediate (IV) underwent cyclization with stirring overnight with an equivalent amount of various substituted 2-bromoacetophenone in EtOH and Et_3_N (catalyst) in order to produce the benzimidazole-oxazole hybrid compounds (**1**–**19**) in proper yield (Scheme 1). After cooling to 25 °C, the solvent was withdrawn, and the resulting solid residue was cleaned by washing with *n*-hexane before being re-crystallized from ethyl alcohol. Many spectroscopic techniques, including ^13^C-NMR, ^1^H-NMR, and HR-EIMS, were used to determine the structures of all freshly synthesized analogues.

### 2.2. In Vitro Acetylcholinesterase and Butyrylcholinesterase Inhibitory Activities

**Figure 5:**
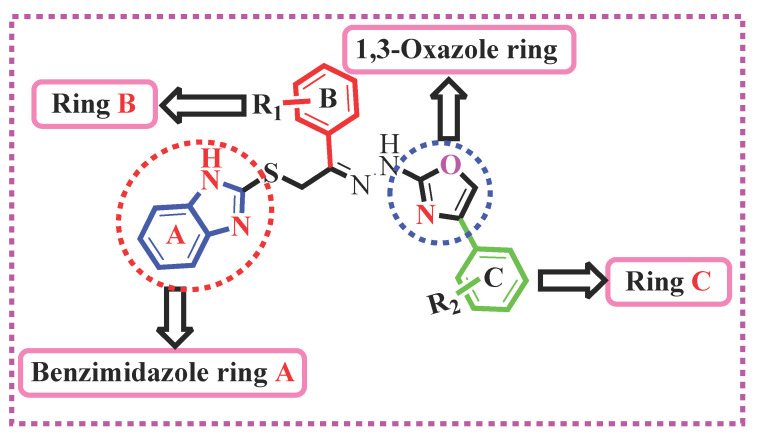
General structure of benzimidazole-oxazole hybrid derivatives (**1**–**19**).

Hybrid analogues based on benzimidazole incorporating an oxazole skeleton were synthesised and then screened in vitro for their inhibitory activities against targeted AChE and BuChE enzymes as compared to donepezil as the standard drug. All synthesised benzimidazole-based oxazole analogues showed a varied range of inhibitory potentials against targeted AChE and BuChE enzymes, with IC50 values ranging from 0.10 ± 0.050 to 12.60 ± 0.30 µM (against AChE) and 0.20 ± 0.050 µM to 16.30 ± 0.30 µM (against BuChE) when compared to standard donepezil (IC50 = 2.16 ± 0.12 µM and 4.5 ± 0.11 µM, respectively (Table 1). Structure-activity relationship (SAR) studies have shown that inhibitory potentials were greatly affected by changing either the position of substituent(s) or the nature of substituent(s) around both aryl parts; therefore, to better understand SAR studies, the synthesized analogues were split into various parts, including benzimidazole, oxazole, and aryl parts (R1 and R2), and it was further revealed from SAR studies that each part effectively contributed to inhibitory potentials, and any variation found on both aryl parts (R1 and R2, respectively) may result in different inhibitory potentials against targeted enzymes (Figure 5).

#### Structure Activity Relationship (SAR) for Acetylcholinesterase (AChE) and Butyrylcholinesterase (BuChE) Activities

Structure-activity relationships were performed on the basis of variety in different substitution patterns on both rings B and C, respectively. Derivatives 9 (IC50 = 0.10 ± 0.050 µM and 0.20 ± 0.050 µM), having a –NO_2_ group at the 3-position on ring C and triflouromethyl substitution at the 4-position on ring B, and derivative 14, having dichloro substitution at the 2- and 3-positions on ring C along with a methyl group at the para position on ring B, were identified as the first and second most potent inhibitors of AChE and BuChE enzymes among the synthesized derivatives.

Among the compounds with an unsubstituted ring B, derivative 10 (IC_50_ = 1.40 ± 0.10 µM and 2.10 ± 0.10 µM) had a nitro group at *meta* on ring C and derivative 17 (IC_50_ = 2.50 ± 0.10 µM and 3.50 ± 0.10µM) had a nitro group at *ortho* on ring C. The difference in the potentials of these derivatives may be due to the different positions of the nitro group on ring C.

By comparing the derivatives with a methyl group on ring B, derivative **2** (IC_50_ =1.60 ± 0.10 µM and1.90 ± 0.10 µM) had a –NO_2_ group at the *ortho* position on ring C, derivative **3** (IC_50_ = 4.40 ± 0.10 µM and 5.80 ± 0.10 µM) had a –NO_2_ group at the *meta* position on ring C, derivative **4** (IC_50_ = 9.80 ± 0.20 µM and 11.50 ± 0.20 µM) had methyl at the *para* position on ring C, derivative **5** (IC_50_ = 9.80 ± 0.20 µM and 11.50 ± 0.20 µM) had a methoxy group at the *meta* position on ring C, derivative **6** (IC_50_ =0.40 ± 0.05 µM and 1.10 ± 0.05 µM) had dichloro substitutions at the *meta* and *para* positions on ring C, derivative **8** (IC_50_ = 5.30 ± 0.20 µM and 7.20 ± 0.10 µM) had a methoxy group at the *ortho* position on ring C, derivative **12** (IC_50_ = 0.40 ± 0.05 µM and 1.10 ± 0.05 µM) had a chloro group at the *para* position on ring C, and derivative **14** (IC_50_ = 0.20 ± 0.050 µM and 0.30 ± 0.050 µM) had dichloro substitutions at the *ortho* and *para* positions on ring C. The difference in the potentials of these entire derivatives may be due to the different nature, number, and position of the substituents on ring C.

Among the derivatives having a chloro group on ring B, compound **11** (IC_50_ = 0.70 ± 0.50 µM and 1.20 ± 0.010 µM) had a NO_2_ group at the *meta* position on ring C, **18** (IC_50_ = 0.80 ± 0.10 µM and 1.70 ± 0.10 µM) had a nitro group at the *ortho* position on ring C, and **19** (IC_50_ = 2.70 ± 0.20 µM and 1.10 ± 0.05 µM) had an OCH_3_ group at the meta position on ring C, correspondingly. The difference in the inhibitory potentials of these derivatives may be due to the different nature, number, and position of the substituents on ring C.

Overall, it was also observed that the variation in number, nature, and position of certain groups at particular positions on rings B and C greatly affects the inhibitory potentials of the synthesized derivatives. Therefore, a molecular docking study was performed to understand the binding interaction of the most potent derivatives with the active site of enzymes.

### 2.3. Molecular Docking Study

**Figure 6:**
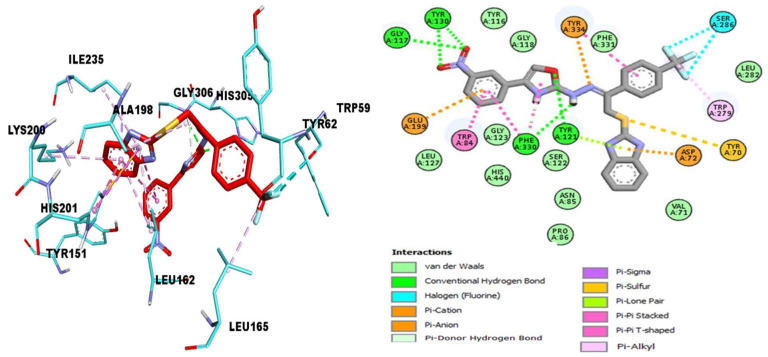
The protein-ligand interaction (PLI) profile of compound **9** against the AChE enzyme and its 3D (**left**) and 2D (**right**) diagrams.

**Figure 7:**
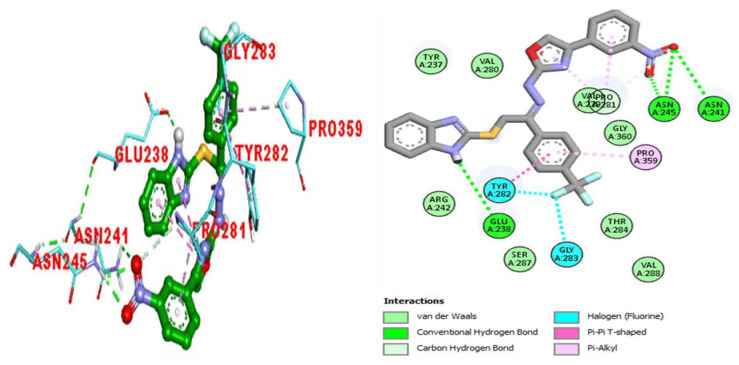
The protein-ligand interaction (PLI) profile of compound **9** against the BuChE enzyme and its 3D (**left**) and 2D (**right**) diagrams.

**Figure 8:**
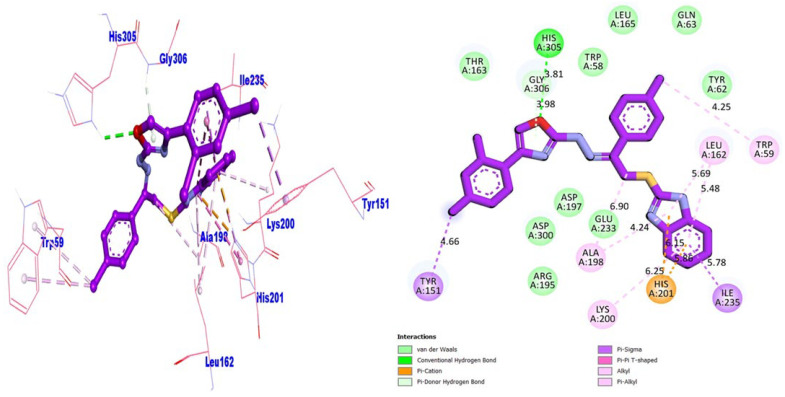
Protein-ligand interaction profile (PLI) of the second most active compound **14** against the AChE enzyme and its 3D (**left**) and 2D (**right**) diagrams.

**Figure 9:**
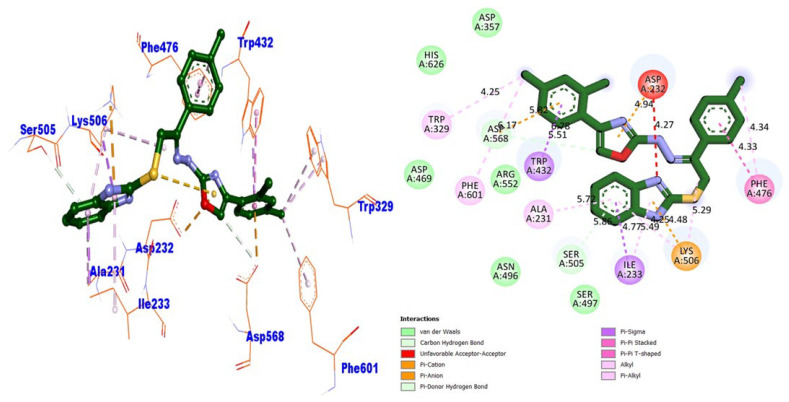
Protein-ligand interaction profile (PLI) of the second most active compound **14** against the BuChE enzyme and its 3D (**left**) and 2D (**right**) diagrams.

**Figure 10:**
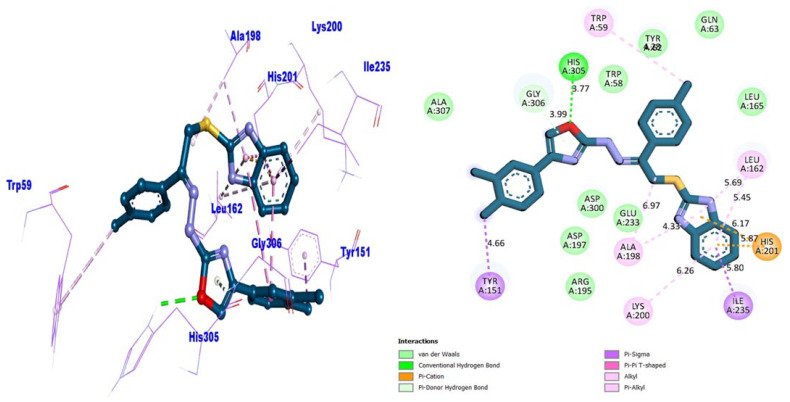
Protein-ligand interaction profile (PLI) of the third most active compound **6** against the AChE enzyme and its 3D (**left**) and 2D (**right**) diagrams.

**Figure 11:**
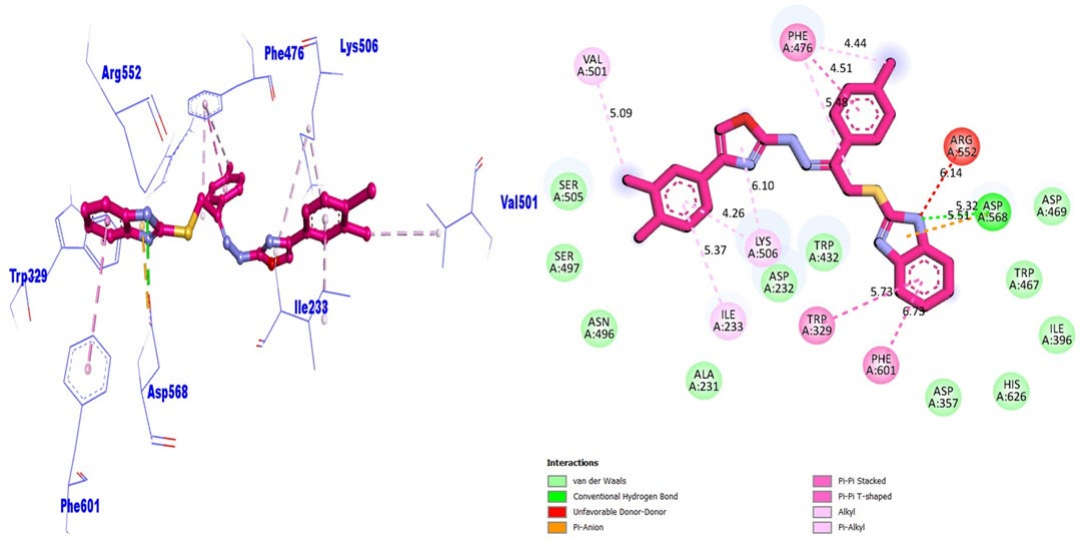
Protein-ligand interaction profile (PLI) of the third most active compound **6** against the BuChE enzyme and its 3D (**left**) and 2D (**right**) diagrams.

A molecular docking study was performed on the most active benzimidazole-oxazole analogues (9, 14, and 6) in order to elucidate the in vitro study. Subsequently, all these active analogues showed a well-fitting binding mode with different binding affinities and correlated well with the in vitro studies. All of the active compounds, 9, 14, and 6, have similar and related chemistry, with slight modifications at different positions. These different functional moieties showed wide differences in their interactions with the active pocket of the targeted enzyme. Compound 9 showed maximum interactions with the active sites of both targeted AChE and BuChE enzymes and was found to be the most effective, and this was similar in the in vitro study. The details of the interactions are given in Table 2. It was also found that not only does the direct attachment of the electron-withdrawing group (–NO_2_) cause good activity, but the indirect attachment of the electron-withdrawing –F_3_ moiety in the form of –CF_3_ is also important, which in turn attaches the ring of the analogue. The better interactions, activity, and docking score of analogue 9 were mainly due to this reason. The electron-withdrawing –NO_2_ and –CF_3_ groups withdraw electronic density from the extended benzene ring, making the benzene ring more electrophilic; hence, the extended benzene ring remains electron-deficient, which further regains stability by forming pi-cation interactions with the active site of the targeted enzyme. From docking analysis, it was found that not only do electron-withdrawing groups such as –NO_2_ and –CF_3_ groups enhance the activity by making the benzene ring cationic, but they also enhance the enzymatic activity through the sidewise involvement of the oxygen of –NO_2_ and –F in hydrogen bonding and halogen (fluorine) with the active sites of both targeted enzymes. The presence of the –NO_2_ functional group is more effective in its *meta*-position; therefore, compound 9 has very good activity and interactions, as shown in Table 2. Compound 14, which holds electron-withdrawing di-Cl moieties at the 2,4-position of ring C and the para-methyl substation on ring B, was proven to be the second most active analogue and adopted several significant interactions with the active sites of both targeted AChE and BuChE (Table 2). Changing the position of di-Cl moieties around ring C, as in the case of compound **6**, resulted in different enzymatic activities and hence different interactions with the active sites of both targeted AChE and BuChE enzymes (Figure 6, Figure 7, Figure 8, Figure 9, Figure 10 and Figure 11, Table 2).

### 2.4. ADMET Analysis

**Figure 12:**
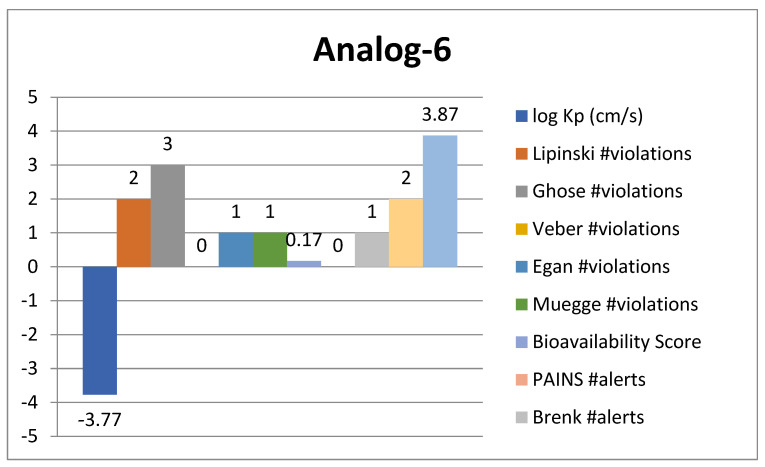
Representation of the ADMET analysis of analog-**6**.

**Figure 13:**
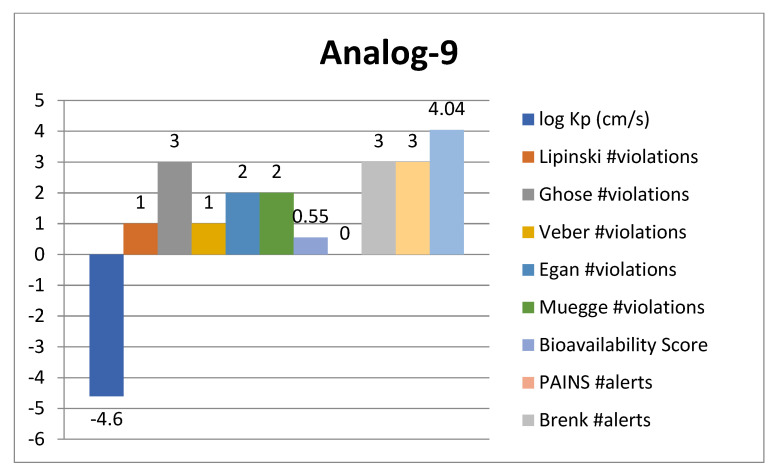
Representation of the ADMET analysis of analog-**9**.

**Figure 14:**
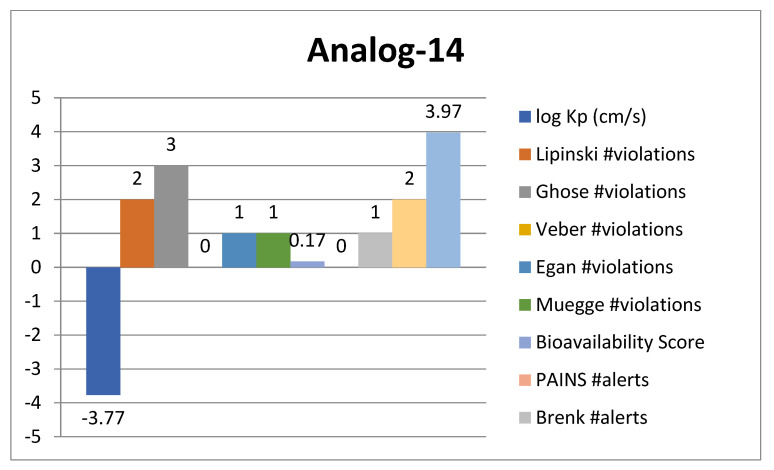
Representation of the ADMET analysis of analog-**14**.

ADMET analysis describes the different properties of the subjected ligand, such as log Kp, Lipinski, Ghose, Veber, Egan, Muegge, Bioavailability, PAINS, and Brenk, etc. (Figure 12, Figure 13 and Figure 14).

## 3. Experiment

### 3.1. General Procedure for the Synthesis of Benzimidazole-Oxazole Hybrid Derivatives (**1**–**19**)

2-Marcaptobenzimidazole (I, 1 mmol) and 2-bromoacetophenone (II, 1 mmol) were mixed and refluxed for 2–3 h in ethanol (10 mL) in the presence of triethylamine to produce the first intermediate product (III) [32]. The second intermediate product (IV) was produced by treating the intermediate (III) and semicarbazide in equal amounts in ethanol (10 mL) and glacial acetic acid as catalysts and refluxing the mixture for around 4 h. After the reaction was complete, the mixture was cooled to room temperature, and the precipitate solid that was produced was filtered and washed with *n*-hexane. TLC plates were used to check the reaction’s development. An equivalent quantity of intermediate (IV) underwent cyclization with stirring overnight with an equivalent amount of various substituted 2-bromoacetophenone in EtOH and Et_3_N (catalyst) in order to produce the benzimidazole-oxazole hybrid compounds (**1**–**19**) at proper yield. After cooling to 25 °C, the solvent was withdrawn, and the resulting solid residue was cleaned by washing with *n*-hexane before being re-crystallized from ethyl alcohol.

### 3.2. Spectral Analysis

Spectra of some compounds are provided as Supplementary Materials.

#### 3.2.1. (E)-2-(2-(2-((1H-Benzo[d]imidazol-2-yl)thio)-1-(4-methoxyphenyl)ethylidene)hydrazinyl)-4-(2-nitrophenyl)oxazole (1)

Yield: 69%; Rf value 0.59 (7:3 n-hex: EtOAc); ^1^ H NMR (600 MHz, DMSO-*d_6_*): δ12.75 (s, 1H, -NH), 10.67 (s, 1H, -NH), 8.21 (dd, *J*_(6″, 5″)_ = 8.0Hz, *J*_(6″, 4″)_ = 2.4Hz, 1H, H-6″), 8.13 (d, *J*_(3″, 4″)_ = 7.8 Hz, 1H, H-3″), 8.02 (d, *J*_(2′, 3′/ 6′, 5′)_ = 8.6Hz, 2H, H-2′/H-6′), 7.96 (s, 1H, oxazole-H), 7.93–7.86 (m, 1H, H-5″), 7.82 (t, *J*_(4″/5″, 3″)_ = 9.0Hz, 1H, H-4″), 7.65 (m, 2H, H-4/H-7), 7.34 (m, 2H, H-5/H-6), 7.13 (d, *J*_(3′, 2′/5′, 6′)_ = 8.0 Hz, 2H, H-3′/H-5′), 3.98 (s, 2H, S-CH_2_), 3.85 (s, 3H, -OCH_3_); ^13^C-NMR (150 MHz, DMSO-d6): δ 163.2, 157.2, 152.2, 149.8, 148.7, 141.7, 141.5, 140.5, 140.3, 136.3, 133.6, 130.6, 129.0, 129.0, 126.6, 126.2, 125.4, 124.6, 124.3, 116.8, 116.6, 114.7, 114.7, 56.1, 39.1.; HR-MS (ESI): (*m*/*z*) [M + H]^+^ calculated for C_25_H_21_N_6_O_4_S 501.1342, found 501.1351.

#### 3.2.2. (*E*)-2-(2-(2-((1H-Benzo[d]imidazol-2-yl)thio)-1-(*p*-tolyl)ethylidene)hydrazinyl)-4-(2-nitrophenyl)oxazole (**2**)

Yield: 67%; Rf value 0.61 (7:3 n-hex: EtOAc); ^1^ H NMR (600 MHz, DMSO-*d_6_*): δ13.34 (s, 1H, -NH), 11.25 (s, 1H, -NH), 7.79 (dd, *J*_(6″, 5″)_ = 8.4 Hz, *J*_(6″, 4″)_ = 2.0 Hz, 1H, H-6″), 7.72 (d, *J*_(3″, 4″)_ = 7.9 Hz, 1H, H-3″), 7.53 (s, 1H, oxazole-H), 7.36–7.33 (m, 1H, H-5″), 7.29 (d, *J*_(2′, 3′/ 6′, 5′)_ = 7.5 Hz, 2H, H-2′/H-6′), 7.24 (t, *J*_(4″/5″, 3″)_ = 9.2 Hz, 1H, H-4″), 7.22 (d, *J*_(3′, 2′/5′, 6′)_ = 6.7 Hz, 2H, H-3′/H-5′), 7.11–7.08 (m, 2H, H-4/H-7), 6.98–6.96 (m, 2H, H-5/H-6), 2.50 (s, 2H, S-CH_2_), 1.22 (s, 3H, -CH_3_); ^13^C-NMR (150 MHz, DMSO-d6): δ 156.6, 151.6, 149.5, 148.1, 141.9, 141.1, 140.9, 139.9, 139.7, 136.0, 133.3, 132.4, 130.5, 130.3, 130.3, 128.2, 128.2, 125.9, 125.1, 124.0, 123.7, 116.2, 116.0, 38.5, 22.5.; HR-MS (ESI): (*m*/*z*) [M + H]^+^ calculated for C_25_H_21_N_6_O_3_S 485.1393, found 485.1401.

#### 3.2.3. (*E*)-2-(2-(2-((1H-Benzo[d]imidazol-2-yl)thio)-1-(*p*-tolyl)ethylidene)hydrazinyl)-4-(3-nitrophenyl)oxazole (**3**)

Yield: 73%; Rf value 0.57 (7:3 n-hex: EtOAc); ^1^ H NMR (600 MHz, DMSO-*d_6_*): δ12.69 (s, 1H, -NH), 10.61 (s, 1H, -NH), 8.53 (t, *J*_(2″/4″, 6″)_ = 2.6 Hz, 1H, H-2″), 8.33 (d, *J*_(6″, 5″)_ = 6.6 Hz, 1H, H-6″), 8.22–8.16 (m, 1H, H-4″), 7.89 (t, *J*_(5″/4″, 6″)_ = 7.8 Hz, 1H, H-5″), 7.84 (s, 1H, oxazole-H), 7.77 (d, *J*_(2′, 3′/ 6′, 5′)_ = 8.2 Hz, 2H, H-2′/H-6′), 7.59–7.51 (m, 2H, H-4/H-7), 7.34 (d, *J*_(3′, 2′/5′, 6′)_ = 7.5 Hz, 2H, H-3′/H-5′), 7.28–7.19 (m, 2H, H-5/H-6), 3.92 (s, 2H, S-CH_2_), 2.49 (s, 3H, -CH_3_).; ^13^C-NMR (150 MHz, DMSO-d6): δ 157.1, 152.1, 149.0, 148.6, 142.4, 141.6, 141.4, 140.4, 140.2, 134.5, 134.2, 132.7, 131.2, 130.8, 130.8, 128.7, 128.7, 124.7, 124.5, 124.2, 123.3, 116.7, 116.5, 39.0, 23.0.; HR-MS (ESI): (*m*/*z*) [M + H]^+^ calculated for C_25_H_21_N_6_O_3_S 485.1393, found 485.1401.

#### 3.2.4. (*E*)-2-(2-(2-((1H-Benzo[d]imidazol-2-yl)thio)-1-(*p*-tolyl)ethylidene)hydrazinyl)-4-(*p*-tolyl)oxazole (**4**)

Yield: 74%; Rf value 0.63 (7:3 n-hex: EtOAc); ^1^ H NMR (600 MHz, DMSO-*d_6_*): δ12.66 (s, 1H, -NH), 10.58 (s, 1H, -NH), 7.83 (d, *J*_(2′, 3′/ 6′, 5′)_ = 6.6 Hz, 2H, H-2′/H-6′), 7.81 (s, 1H, oxazole-H), 7.69 (d, *J*_(2″, 3″/ 6″, 5″)_ = 7.9 Hz, 2H, H-2″/H-6″), 7.56–7.48 (m, 2H, H-4/H-7), 7.40 (d, *J*_(3′, 2′/5′, 6′)_ = 7.0 Hz, 2H, H-3′/H-5′), 7.29–7.22 (m, 2H, H-5/H-6), 7.23 (d, *J*_(3″, 2″/5″, 6″)_ = 8.5 Hz, 2H, H-3″/H-5″), 3.89 (s, 2H, S-CH_2_), 2.44 (s, 3H, -CH_3_), 2.54 (s, 3H, -CH_3_).; ^13^C-NMR (150 MHz, DMSO-d6): δ 165.9, 164.3, 162.7, 160.3,159.4, 157.07, 135.9, 131.0, 131.0, 130.6, 130.6, 130.2, 130.1,129.4, 129.3, 128.6, 128.3, 128.2, 127.7, 120.6, 116.1, 115.9, 115.8, 115.8, 115.7, 64.8, 40.0, 21.0, 15.1.; HR-MS (ESI): (*m*/*z*) [M + H]^+^ calculated for C_26_H_24_N_5_OS 454.1698, found 454.1707.

#### 3.2.5. (*E*)-2-(2-(2-((1H-Benzo[d]imidazol-2-yl)thio)-1-(*p*-tolyl)ethylidene)hydrazinyl)-4-(3-methoxyphenyl)oxazole (**5**)

Yield: 76%; Rf value 0.56 (7:3 n-hex: EtOAc); ^1^ H NMR (600 MHz, DMSO-*d_6_*): δ12.80 (s, 1H, -NH), 10.72 (s, 1H, -NH), 7.95 (s, 1H, oxazole-H), 7.76–7.69 (m, 2H, H-4/H-7), 7.65 (d, *J*_(2′, 3′/ 6′, 5′)_ = 9.4 Hz, 2H, H-2′/H-6′), 7.60–7.54 (m, 1H, H-6″), 7.52 (dd, *J*_(5″, 4″)_ = 9.2 Hz, *J*_(5″, 6″)_ = 9.0 Hz, 1H, H-5″), 7.44 (t, *J*_(2″/4″, 6″)_ = 2.8 Hz, 1H, H-2″), 7.39 (m, 2H, H-5/H-6), 7.18 (d, *J*_(3′, 2′/5′, 6′)_ = 8.6 Hz, 2H, H-3′/H-5′), 7.14 (d, *J*_(4″, 5″)_ = 8.5 Hz, 1H, H-4″), 4.04 (s, 2H, S-CH_2_), 3.91 (s, 3H, -OCH_3_), 2.32 (s, 3H, -CH_3_).; ^13^C-NMR (150 MHz, DMSO-d6): δ 161.7, 155.2, 150.2, 146.7, 142.1, 139.7, 139.5, 138.5, 138.3, 134.6, 132.4, 130.8, 130.5, 130.5, 128.4, 128.4, 122.6, 122.3, 120.4, 114.9, 114.8, 114.6, 114.2, 56.4, 37.1, 22.7; HR-MS (ESI): (*m*/*z*) [M + H]^+^ calculated for C_26_H_24_N_5_O_2_S 470.1645, found 470.1656.

#### 3.2.6. (*E*)-2-(2-(2-((1H-Benzo[d]imidazol-2-yl)thio)-1-(*p*-tolyl)ethylidene)hydrazinyl)-4-(3,4-dichlorophenyl)oxazole (**6**)

Yield: 67%; Rf value 0.58 (7:3 n-hex: EtOAc); ^1^ H NMR (600 MHz, DMSO-*d_6_*): δ12.80 (s, 1H, -NH), 10.72 (s, 1H, -NH), 8.09 (d, *J*_(2″, 6″)_ = 2.8 Hz, 1H, H-2″), 7.98 (dd, *J*_(6″, 5″)_ = 8.8 Hz, *J*_(6″, 2″)_ = 1.7 Hz, 1H, H-6″), 7.93 (s, 1H, oxazole-H), 7.81 (d, *J*_(2′, 3′/ 6′, 5′)_ = 8.4 Hz, 2H, H-2′/H-6′), 7.75–7.70 (m, 2H, H-4/H-7), 7.65 (d, *J*_(5″, 6″)_ =7.5 Hz, 1H, H-5″), 7.45–7.39 (m, 2H, H-5/H-6), 7.33 (d, *J*_(3′, 2′/5′, 6′)_ = 9.0 Hz, 2H, H-3′/H-5′), 4.04 (s, 2H, S-CH_2_), 2.48 (s, 3H, -CH_3_).; ^13^C-NMR (150 MHz, DMSO-d6): δ 157.6, 152.6, 149.1, 142.4, 142.1, 141.9, 140.9, 140.7, 134.4, 133.7, 133.5, 132.5, 131.7, 130.6, 130.6, 129.8, 128.5, 128.5, 128.0, 125.0, 124.7, 117.2, 117.0, 39.5, 22.8.; HR-MS (ESI): (*m*/*z*) [M + H]^+^ calculated for C_25_H_20_Cl_2_N_5_OS 508.0763, found 508.0771.

#### 3.2.7. (*E*)-4-(2-((1H-Benzo[d]imidazol-2-yl)thio)-1-(2-(4-(4-bromophenyl)oxazol-2-yl)hydrazono)ethyl)-*N*,*N*-dimethylaniline (**7**)

Yield: 65%; Rf value 0.53 (7:3 n-hex: EtOAc); ^1^ H NMR (600 MHz, DMSO-*d_6_*): δ13.33 (s, 1H, -NH), 11.26 (s, 1H, -NH), 7.84 (s, 1H, oxazole-H), 7.77 (d, *J*_(2″, 3″/ 6″, 5″)_ = 7.9 Hz, 2H, H-2″/H-6″), 7.55–7.53 (m, 2H, H-4/H-7), 7.45 (d, *J*_(3″, 2″/5″, 6″)_ = 7.5 Hz, 2H, H-3″/H-5″), 7.36 (d, *J*_(2′, 3′/ 6′, 5′)_ = 8.6 Hz, 2H, H-2′/H-6′), 7.33 (m, 2H, H-5/H-6), 7.10–6.84 (d, *J*_(3′, 2′/5′, 6′)_ = 9.4 Hz, 2H, H-3′/H-5′), 3.81 (s, 6H, -N(CH_3_)_2_), 3.51 (s, 2H, S-CH_2_); ^13^C-NMR (150 MHz, DMSO-d6): δ 155.3, 150.3, 146.8, 142.6, 139.8, 139.6, 138.6, 138.4, 132.9, 132.5, 132.5, 131.0, 131.0, 130.1, 128.9, 128.9, 128.7, 128.7, 123.6, 122.7, 122.4, 114.9, 114.7, 37.2, 23.2.; HR-MS (ESI): (*m*/*z*) [M + H]^+^ calculated for C_26_H_24_BrN_6_OS 547.0911, found 547.0908.

#### 3.2.8. (*E*)-2-(2-(2-((1H-Benzo[d]imidazol-2-yl)thio)-1-(*p*-tolyl)ethylidene)hydrazinyl)-4-(2-methoxyphenyl)oxazole (**8**)

Yield: 78%; Rf value 0.56 (7:3 n-hex: EtOAc); ^1^ H NMR (600 MHz, DMSO-*d_6_*): δ12.64 (s, 1H, -NH), 10.56 (s, 1H, -NH), 8.33 (d, *J*_(6″, 5″)_ = 7.0 Hz, 1H, H-6″), 7.83 (d, *J*_(2′, 3′/ 6′, 5′)_ = 7.7 Hz, 2H, H-2′/H-6′), 7.75 (s, 1H, oxazole-H), 7.55 (m, 2H, H-4/H-7), 7.52 (t, *J*_(4″/5″, 3″)_ = 9.0 Hz, 1H, H-4″), 7.47 (dd, *J*_(5″, 4″)_ = 8.8 Hz, *J*_(5″, 6″)_ = 7.9 Hz, 1H, H-5″), 7.37 (d, *J*_(3′, 2′/5′, 6′)_ = 6.8 Hz, 2H, H-3′/H-5′), 7.26 (d, *J*_(3″, 4″)_ = 8.7 Hz, 1H, H-3″), 7.23 (m, 2H, H-5/H-6), 3.87 (s, 2H, S-CH_2_), 3.82 (s, 3H, -OCH_3_), 2.53 (s, 3H, -CH_3_).; ^13^C-NMR (150 MHz, DMSO-d6): δ 158.3, 156.9, 151.9, 148.4, 141.4, 141.2, 140.2, 142.0, 140.0, 132.5, 132.1, 130.7, 130.4, 130.4, 128.3, 128.3, 124.3, 124.0, 122.5, 119.9, 116.5, 116.3, 112.1, 57.1, 38.8, 22.6.; HR-MS (ESI): (*m*/*z*) [M + H]^+^ calculated for C_26_H_24_N_5_O_2_S 470.1645, found 470.1656.

#### 3.2.9. (*E*)-4-(2-(2-(2-((1H-Benzo[d]imidazol-2-yl)thio)-1-(*p*-tolyl)ethylidene)hydrazinyl) Oxa-zol-4-yl)phenol (**9**)

Yield: 70%; Rf value 0.64 (7:3 n-hex: EtOAc); ^1^ H NMR (600 MHz, DMSO-*d_6_*): δ12.77 (s, 1H, -NH), 10.69 (s, 1H, -NH), 9.77 (s, 1H, -OH), 7.92 (s, 1H, oxazole-H), 7.68–7.63 (m, 2H, H-4/H-7), 7.58 (d, *J*_(2′, 3′/ 6′, 5′)_ = 9.1 Hz, 2H, H-2′/H-6′), 7.48 (d, *J*_(2″, 3″/ 6″, 5″)_ = 7.0 Hz, 2H, H-2″/H-6″), 7.36–7.29 (m, 2H, H-5/H-6), 7.17 (d, *J*_(3′, 2′/5′, 6′)_ = 8.3 Hz, 2H, H-3′/H-5′), 6.96 (d, *J*_(3″, 2″/5″, 6″)_ = 8.2 Hz, 2H, H-3″/H-5″), 4.01 (s, 2H, S-CH_2_), 2.31 (s, 3H, -CH_3_).; ^13^C-NMR (150 MHz, DMSO-d6): δ 159.5, 155.0, 150.0, 146.5, 142.5, 139.5, 139.3, 138.3, 138.1, 132.8, 130.9, 130.9, 129.9, 129.9, 128.8, 128.8, 124.3, 122.4, 122.1, 117.4, 117.4, 114.6, 114.4, 36.9, 23.1.; HR-MS (ESI): (*m*/*z*) [M + H]^+^ calculated for C_25_H_22_N_5_O_2_S 456.1488, found 456.1499.

#### 3.2.10. (*E*)-2-(2-(2-((1H-Benzo[d]imidazol-2-yl)thio)-1-phenylethylidene)hydrazinyl)-4-(3-nitrophenyl)oxazole (**10**)

Yield: 73%; Rf value 0.57 (7:3 n-hex: EtOAc); ^1^ H NMR (600 MHz, DMSO-*d_6_*): δ12.82 (s, 1H, -NH), 10.74 (s, 1H, -NH), 8.52 (t, *J*_(2″/4″, 6″)_ = 2.5 Hz, 1H, H-2″), 8.34 (d, *J*_(6″, 5″)_ = 9.0 Hz, 1H, H-6″), 8.21–8.17 (m, 1H, H-4″), 8.07–8.01 (m, 2H, H-2′/H-6′), 7.88 (t, *J*_(5″/4″,6″)_ = 8.1 Hz, 1H, H-5″), 7.78 (s, 1H, oxazole-H), 7.72 (m, 2H, H-4/H-7), 7.57–7.51 (m, 3H, H-3′/H-4′/H-5′), 7.41 (m, 2H, H-5/H-6), 4.06 (s, 2H, S-CH_2_).; ^13^C-NMR (150 MHz, DMSO-d6): δ 157.0, 152.0, 148.8, 148.5, 141.5, 141.3, 140.3, 140.1, 136.0, 134.3, 134.0, 133.0, 131.0, 130.8, 130.8, 130.2, 130.2, 124.5, 124.3, 124.0, 123.1, 116.6, 116.4, 38.9.; HR-MS (ESI): (*m*/*z*) [M + H]^+^ calculated for C_24_H_19_N_6_O_3_S 471.1237, found 471.1245.

#### 3.2.11. (*E*)-2-(2-(2-((1H-Benzo[d]imidazol-2-yl)thio)-1-(4-chlorophenyl)ethylidene)hydrazi-nyl)-4-(3-nitrophenyl)oxazole (**11**)

Yield: 79%; Rf value 0.61 (7:3 n-hex: EtOAc); ^1^ H NMR (600 MHz, DMSO-*d_6_*): δ12.68 (s, 1H, -NH), 10.60 (s, 1H, -NH), 8.50 (t, *J*_(2″/4″, 6″)_ = 2.0 Hz, 1H, H-2″), 8.31 (d, *J*_(6″, 5″)_ = 7.7 Hz, 1H, H-6″), 8.19–8.13 (m, 1H, H-4″), 8.08 (d, *J*_(2′, 3′/ 6′, 5′)_ = 9.0 Hz, 2H, H-2′/H-6′), 7.86 (t, *J*_(5″/4″,6″)_ = 7.5Hz, 1H, H-5″), 7.83 (s, 1H, oxazole-H), 7.72 (d, *J*_(3′, 2′/5′, 6′)_ = 8.4 Hz, 2H, H-3′/H-5′), 7.58 (m, 2H, H-4/H-7), 7.27 (m, 2H, H-5/H-6), 3.91 (s, 2H, S-CH_2_).; ^13^C-NMR (150 MHz, DMSO-d6): δ. 166.1, 163.1, 149.8, 41.2, 133.1, 132.2, 131.6, 131.6, 131.5, 130.5, 127.8, 127.6,127.5, 122.4, 122.3, 121.3, 120.9, 119.8, 119.6,111.0, 107.5, 40.0; HR-MS (ESI): (*m*/*z*) [M + H]^+^ calculated for C_24_H_18_ClN_6_O_3_S 508.0846, found 508.0855.

#### 3.2.12. (*E*)-2-(2-(2-((1H-Benzo[d]imidazol-2-yl)thio)-1-(*p*-tolyl)ethylidene)hydrazinyl)-4-(4-chlorophenyl)oxazole (**12**)

Yield: 72%; Rf value 0.65 (7:3 n-hex: EtOAc); ^1^ H NMR (600 MHz, DMSO-*d_6_*): δ12.70 (s, 1H, -NH), 10.62 (s, 1H, -NH), 8.08 (d, *J*_(2″, 3″/ 6″, 5″)_ = 6.9 Hz, 2H, H-2″/H-6″), 7.85 (s, 1H, oxazole-H), 7.75 (d, *J*_(2′, 3′/ 6′, 5′)_ = 8.8 Hz, 2H, H-2′/H-6′), 7.63 (d, *J*_(3″, 2″/5″, 6″)_ = 7.7 Hz, 2H, H-3″/H-5″), 7.57–7.50 (m, 2H, H-4/H-7), 7.34 (d, *J*_(3′, 2′/5′, 6′)_ = 9.4 Hz, 2H, H-3′/H-5′), 7.28–7.18 (m, 2H, H-5/H-6), 3.93 (s, 2H, S-CH_2_), 2.47 (s, 3H, -CH_3_).; ^13^C-NMR (150 MHz, DMSO-d6): δ 166.1, 163.0, 161.3, 159.4, 156.7, 153.5, 136.9, 136.8, 135.7, 130.9, 130.9, 130.8, 129.4, 129.3, 128.8, 128.3, 127.9, 127.7, 126.2, 124.2, 122.2, 120.6, 117.5, 117.4, 115.7, 113.8, 113.7, 64.8, 15.1; HR-MS (ESI): (*m*/*z*) [M + H]^+^ calculated for C_25_H_21_N_6_O_3_S 485.1393, found 485.1401.

#### 3.2.13. (*E*)-2-(2-(2-((1H-Benzo[d]imidazol-2-yl)thio)-1-(*p*-tolyl)ethylidene)hydrazinyl)-4-phenyloxazole (**13**)

Yield: 68%; Rf value 0.54 (7:3 n-hex: EtOAc); ^1^ H NMR (600 MHz, DMSO-*d_6_*): δ12.79 (s, 1H, -NH), 10.71 (s, 1H, -NH), 7.97 (s, 1H, oxazole-H), 7.91–7.85 (m, 2H, H-2″/H-6″), 7.80 (d, *J*_(2′, 3′/ 6′, 5′)_ = 7.1 Hz, 2H, H-2′/H-6′), 7.74–7.68 (m, 2H, H-4/H-7), 7.63–7.56 (m, 3H, H-3″/H-4″/H-5″), 7.46 (d, *J*_(3′, 2′/5′, 6′)_ = 6.9 Hz, 2H, H-3′/H-5′), 7.38–7.31 (m, 2H, H-5/H-6), 4.03 (s, 2H, S-CH_2_), 2.54 (s, 3H, -CH_3_).; ^13^C-NMR (150 MHz, DMSO-d6): δ 155.5, 150.5, 147.0, 143.4, 140.0, 139.8, 138.8, 138.6, 132.7, 131.7, 130.8, 130.8, 130.2, 130.2, 129.7, 128.7, 128.7, 125.7, 125.7, 122.9, 122.6, 115.1, 114.9, 37.4, 22.8.; HR-MS (ESI): (*m*/*z*) [M + H]^+^ calculated for C_25_H_21_ClN_5_OS 474.1151, found 474.1161.

#### 3.2.14. (*E*)-2-(2-(2-((1H-Benzo[d]imidazol-2-yl)thio)-1-(*p*-tolyl)ethylidene)hydrazinyl)-4-(2,4-dichlorophenyl)oxazole (**14**)

Yield: 80%; Rf value 0.57 (7:3 n-hex: EtOAc); ^1^ H NMR (600 MHz, DMSO-*d_6_*): δ12.77 (s, 1H, -NH), 10.69 (s, 1H, -NH), 8.13 (d, *J*_(6″, 5″)_ =8.8 Hz, 1H, H-6″), 7.92 (s, 1H, oxazole-H), 7.79 (d, *J*_(2′, 3′/ 6′, 5′)_ = 7.8 Hz, 2H, H-2′/H-6′), 7.67–7.63 (m, 2H, H-4/H-7), 7.59 (d, *J*_(3″, 5″)_ = 1.6 Hz, 1H, H-3″), 7.46 (dd, *J*_(5″, 6″)_ = 8.4 Hz, *J*_(5″, 3″)_ =1.8 Hz, 1H, H-5″), 7.41 (d, *J*_(3′, 2′/5′, 6′)_ = 7.9 Hz, 2H, H-3′/H-5′), 7.36–7.30 (m, 2H, H-5/H-6), 4.01 (s, 2H, S-CH_2_), 2.51 (s, 3H, -CH_3_).; ^13^C-NMR (150 MHz, DMSO-d6): δ 157.5, 152.5, 149.0, 143.3, 142.0, 141.8, 140.8, 140.6, 136.7, 134.6, 132.6, 131.9, 131.3, 130.7, 130.7, 129.0, 128.6, 128.6, 128.4, 124.9, 124.6, 117.1, 116.9, 39.4, 22.9.; HR-MS (ESI): (*m*/*z*) [M + H]^+^ calculated for C_25_H_20_Cl_2_N_5_OS 508.0763, found 508.0771.

#### 3.2.15. (*E*)-2-(2-(2-((1H-Benzo[d]imidazol-2-yl)thio)-1-phenylethylidene)hydrazinyl)-4-(3-methoxyphenyl)oxazole (**15**)

Yield: 74%; Rf value 0.60 (7:3 n-hex: EtOAc); ^1^ H NMR (600 MHz, DMSO-*d_6_*): δ12.71 (s, 1H, -NH), 10.63 (s, 1H, -NH), 7.86–7.79 (m, 2H, H-2′/H-6′), 7.73 (s, 1H, oxazole-H), 7.69–7.64 (m, 1H, H-6″), 7.60 (m, 2H, H-4/H-7), 7.51 (dd, *J*_(5″, 4″)_ = 9.0 Hz, *J*_(5″, 6″)_ = 9.3 Hz, 1H, H-5″), 7.47–7.38 (m, 3H, H-3′/H-4′/H-5′), 7.36 (t, *J*_(2″/4″, 6″)_ = 2.6Hz, 1H, H-2″), 7.30 (m, 2H, H-5/H-6), 7.13 (d, *J*_(4″, 5″)_ = 8.4 Hz, 1H, H-4″), 3.95 (s, 2H, S-CH_2_), 3.89 (s, 3H, -OCH_3_).; ^13^C-NMR (150 MHz, DMSO-d6): δ 161.6, 155.1, 150.1, 146.6, 139.6, 139.4, 138.4, 138.2, 136.5, 134.5, 133.5, 131.3, 131.3, 130.9, 130.7, 130.7, 122.5, 122.2, 120.3, 114.9, 114.7, 114.5, 114.1, 56.3, 37.0.; HR-MS (ESI): (*m*/*z*) [M + H]^+^ calculated for C_25_H_22_N_5_O_2_S 456.1487, found 456.1499.

#### 3.2.16. (*E*)-2-(2-(2-((1H-Benzo[d]imidazol-2-yl)thio)-1-([1,1′-biphenyl]-4-yl)ethylidene) Hydrazinyl)-4-(*p*-tolyl)oxazole (**16**)

Yield: 67%; Rf value 0.52 (7:3 n-hex: EtOAc); ^1^ H NMR (600 MHz, DMSO-*d_6_*): δ11.27 (s, 1H, -NH), 10.04 (s, 1H, -NH), 8.27 (d, *J*_(2′, 3′/ 6′, 5′)_ = 7.9 Hz, 2H, H-2′/H-6′), 8.15 (d, *J*_(3′, 2′/5′, 6′)_ = 8.5 Hz, 2H, H-3′/H-5′), 7.99 (s, 1H, oxazole-H), 7.63 (d, *J*_(2″, 3″/ 6″, 5″)_ = 8.7 Hz, 2H, H-2″/H-6″), 7.54 (d, *J*_(3″, 2″/5″, 6″)_ = 8.1 Hz, 2H, H-3″/H-5″), 7.35–7.03 (m, 2H, H-4/H-7), 6.97–6.89 (m, 5H, *para*-Ph), 6.77–6.75 (m, 2H, H-5/H-6), 2.50 (s, 2H, S-CH_2_), 1.23 (s, 3H, -CH_3_).; ^13^C-NMR (150 MHz, DMSO-d6): δ 156.8, 151.8, 148.3, 143.6, 141.6, 141.3, 141.1, 140.1, 139.9, 133.3, 132.1, 129.9, 129.9, 129.7, 129.7, 129.5, 129.5, 128.5, 128.5, 128.1, 127.8, 127.7, 127.7, 126.1, 126.1, 124.2, 123.9, 116.4, 116.2, 38.7, 21.7.; HR-MS (ESI): (*m*/*z*) [M + H]^+^ calculated for C_31_H_26_N_5_OS 485.1854, found 516.1863.

#### 3.2.17. (*E*)-2-(2-(2-((1H-Benzo[d]imidazol-2-yl)thio)-1-phenylethylidene)hydrazinyl)-4-(2-nitrophenyl)oxazole (**17**)

Yield: 81%; Rf value 0.62 (7:3 n-hex: EtOAc); ^1^ H NMR (600 MHz, DMSO-*d_6_*): δ12.67 (s, 1H, -NH), 10.59 (s, 1H, -NH), 8.57 (t, *J*_(2″/4″, 6″)_ = 2.8Hz, 1H, H-2″), 8.38 (d, *J*_(6″, 5″)_ = 6.6Hz, 1H, H-6″), 8.27–8.19 (m, 1H, H-4″), 7.99–7.94 (m, 2H, H-2′/H-6′), 7.91 (t, *J*_(5″/4″,6″)_ = 7.8Hz, 1H, H-5″), 7.82 (s, 1H, oxazole-H), 7.57–7.51 (m, 2H, H-4/H-7), 7.48–7.39 (m, 3H, H-3′/H-4′/H-5′), 7.26–7.18 (m, 2H, H-5/H-6), 3.90 (s, 2H, S-CH_2_).; ^13^C-NMR (150 MHz, DMSO-d6): δ 157.7, 152.7, 149.4, 149.2, 142.2, 142.0, 141.0, 140.8, 136.3, 136.1, 133.2, 133.0, 131.0, 131.0, 130.7, 130.5, 130.3, 125.6, 125.4, 125.1, 124.8, 117.3, 117.1, 39.6.; HR-MS (ESI): (*m*/*z*) [M + H]^+^ calculated for C_24_H_19_N_6_O_3_S 471.1237, found 471.1245.

#### 3.2.18. (*E*)-2-(2-(2-((1H-Benzo[d]imidazol-2-yl)thio)-1-(4-chlorophenyl)ethylidene)hydrazinyl)-4-(2-nitrophenyl)oxazole (**18**)

Yield: 83%; Rf value 0.58 (7:3 n-hex: EtOAc); ^1^ H NMR (600 MHz, DMSO-*d_6_*): δ12.74 (s, 1H, -NH), 10.66 (s, 1H, -NH), 8.52 (t, *J*_(2″/4″, 6″)_ = 1.8Hz, 1H, H-2″), 8.31 (d, *J*_(6″, 5″)_ = 6.9 Hz, 1H, H-6″), 8.24–8.17 (m, 1H, H-4″), 8.09 (d, *J*_(2′, 3′/ 6′, 5′)_ = 9.5 Hz, 2H, H-2′/H-6′),7.90 (t, *J*_(5″/4″,6″)_ = 8.6 Hz, 1H, H-5″), 7.87 (s, 1H, oxazole-H), 7.77 (d, *J*_(3′, 2′/5′, 6′)_ = 8.6 Hz, 2H, H-3′/H-5′), 7.64–7.56 (m, 2H, H-4/H-7), 7.33–7.25 (m, 2H, H-5/H-6), 3.97 (s, 2H, S-CH_2_).; ^13^C-NMR (150 MHz, DMSO-d6): δ 157.8, 152.8, 149.3, 149.1, 142.3, 141.1, 141.1, 140.9, 138.4, 136.1, 133.5, 133.0, 130.8, 130.8, 130.6, 130.1, 130.1, 125.8, 125.4, 125.2, 124.9, 117.4, 117.2, 39.7.; HR-MS (ESI): (*m*/*z*) [M + H]^+^ calculated for C_24_H_18_ClN_6_O_3_S 508.0846, found 508.0855.

#### 3.2.19. (*E*)-2-(2-(2-((1H-Benzo[d]imidazol-2-yl)thio)-1-(4-chlorophenyl)ethylidene)hydrazinyl)-4-(3-methoxyphenyl)oxazole (**19**)

Yield: 79%; Rf value 0.56 (7:3 n-hex: EtOAc); ^1^ H NMR (600 MHz, DMSO-*d_6_*): δ12.78 (s, 1H, -NH), 10.70 (s, 1H, -NH), 8.07 (d, *J*_(2′, 3′/ 6′, 5′)_ = 9.2 Hz, 2H, H-2′/H-6′), 7.93 (s, 1H, oxazole-H), 7.74 (d, *J*_(3′, 2′/5′, 6′)_ = 7.9 Hz, 2H, H-3′/H-5′), 7.68 (m, 2H, H-4/H-7), 7.63–7.56 (m, 1H, H-6″), 7.50 (dd, *J*_(5″, 4″)_ = 8.3 Hz, *J*_(5″, 6″)_ = 8.9 Hz, 1H, H-5″), 7.39 (t, *J*_(2″/4″, 6″)_ = 1.8Hz, 1H, H-2″), 7.34–7.28 (m, 2H, H-5/H-6), 7.12 (d, *J*_(4″, 5″)_ = 7.5 Hz, 1H, H-4″), 4.02 (s, 2H, S-CH_2_), 3.89 (s, 3H, -OCH_3_).; ^13^C-NMR (150 MHz, DMSO-d6): δ 161.9, 157.3, 152.3, 148.8, 141.8, 141.6, 140.6, 140.4, 138.7, 134.7, 133.2, 131.0, 131.0, 130.9, 130.3, 130.3, 124.7, 124.4, 120.6, 116.9, 116.7, 115.0, 114.4, 56.7, 39.2.; HR-MS (ESI): (*m*/*z*) [M + H]^+^ calculated for C_25_H_21_ClN_5_O_2_S 490.1098, found 490.1109.

### 3.3. Assay Protocol for Acetylcholinesterase and Butyrylcholinesterase Activities

According to previously established protocols, in vitro AChE and BuChE inhibition profiles were evaluated with slight modifications [33,34].

### 3.4. Protocol for Molecular Docking Study

In order to properly triangulate the in vitro and in silico results, a molecular docking analysis was carried out using the AutoDock Vina software (version 1.5.7) package to understand the binding mode of synthetic compounds against both the targeted AChE and BuChE enzymes. The RCSB protein databank’s crystal structures for both targets were obtained using the PDB codes 1ACL for AChE and 1P0P for BuChE. The crystallographic structures and all of the produced compounds were protonated and energy was reduced using the default MOE-Dock module parameters, resulting in optimized enzyme and compound structures. Docking research was then conducted using the improved enzyme and chemical structures. Our prior investigations covered all of the docking protocol’s specifics in depth [35,36].

## 4. Conclusions

In conclusion, we synthesized benzimidazole-based oxazole derivatives (1–19) and evaluated them against both AChE and BuChE enzymes, respectively. All of the synthesized analogues were evaluated for their inhibition profiles against the AChE and BuChE enzymes as compared to donepezil, the standard drug. All of the synthesized benzimidazole-based oxazole analogues showed a varied range of inhibitory potentials against targeted AChE and BuChE enzymes, with IC_50_ values ranging from 0.10 ± 0.050 to 12.60 ± 0.30 µM (for AChE) and 0.20 ± 0.050 µM to 16.30 ± 0.30 µM (for BuChE) when compared to standard donepezil (IC_50_ = 2.16 ± 0.12 µM for AChE) and 4.50 ± 0.11 µM for BuChE). Structure-activity relationship (SAR) studies revealed that analogues 9 (bearing *para*-CF_3_ on ring B and *meta*-NO_2_ substitution at ring C) and 14 (that holds *para*-CH_3_ substitutions on ring B and 2,4-diCl moieties on ring C) were the most potent analogues of the AChE and BuChE enzymes, with IC_50_ values of 0.10 ± 0.050 and 0.20 ± 0.050 µM (against AChE) and 0.20 ± 0.050 and 0.30 ± 0.050 µM (against BuChE), respectively. In order to explore the binding interactions possessed by active scaffolds with the active sites of amino acids of the targeted AChE and BuChE enzymes, the active analogues were also subjected to molecular docking studies. The results revealed that these active analogues provided numerous important interactions with the targeted enzymes’ active sites. All of the synthesized analogues were structurally elucidated using spectroscopic methods such as ^1^H-NMR, ^13^C-NMR, and HREI-MS.

## Figures and Tables

**Table 1:** Different substituents, AChE and BuChE activities of benzimidazole-oxazole hybrid derivatives (**1**–**19**).

S.NO	R_1_	R_2_	AChE IC_50_ ± SEM ^a^ (µM)	BuChE IC_50_ ± SEM ^a^ (µM)
1	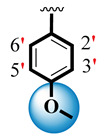	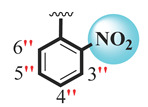	1.10 ± 0.10	1.80 ± 0.10
2	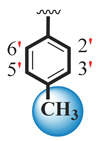	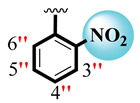	1.60 ± 0.010	1.90 ± 0.10
3	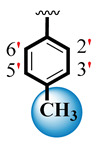	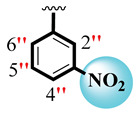	4.40 ± 0.10	5.80 ± 0.10
4	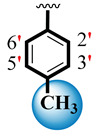	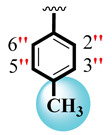	6.50 ± 0.20	8.30 ± 0.20
5	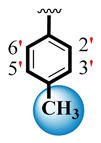	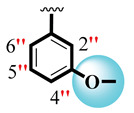	9.80 ± 0.20	11.50 ± 0.20
6	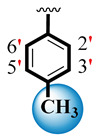	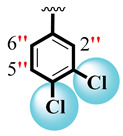	0.40 ± 0.05	1.10 ± 0.05
7	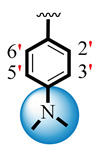	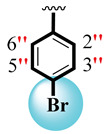	12.60 ± 0.30	16.30 ± 0.30
8	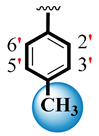	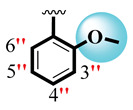	5.30 ± 0.20	7.20 ± 0.10
9	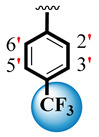	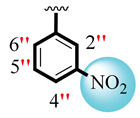	0.10 ± 0.050	0.20 ± 0.05
10	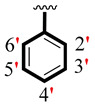	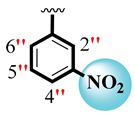	1.40 ± 0.10	2.10 ± 0.10
11	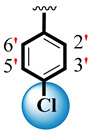	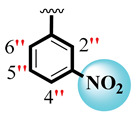	0.70 ± 0.050	1.20 ± 0.010
12	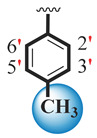	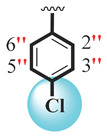	3.40 ± 0.10	5.70 ± 0.10
13	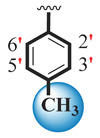	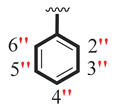	8.10 ± 0.10	9.70 ± 0.10
14	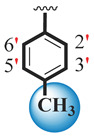	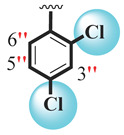	0.20 ± 0.050	0.30 ± 0.050
15	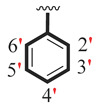	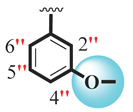	5.40 ± 0.10	7.20 ± 0.10
16	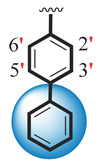	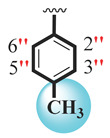	6.10 ± 0.10	9.30 ± 0.10
17	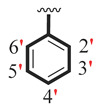	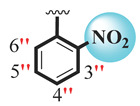	2.50 ± 0.10	3.50 ± 0.10
18	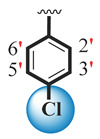	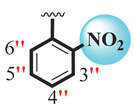	0.80 ± 0.10	1.70 ± 0.10
19	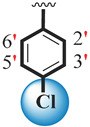	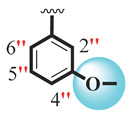	2.70 ± 0.20	3.70 ± 0.10
Standard drug Donepezil			2.16 ± 0.12	4.5 ± 0.11

^a^ SEM is the standard error mean.

**Table 2:** The different types of interactions between active analogues (**9**, **14**, and **6**) and interactive residues of amino acids of the targeted AChE and BuChE enzymes with varied distances.

Active Analogues	Targeted Enzymes	Receptors	Interactions	Binding Affinities (kJ/mol)
Analogue-**9**	AChE	GLY-117	HB	−12.37
		TYR-130	HB	
		GLU-198	π-cation	
		TRP-84	π-π T-shaped	
		PHE-330	π-π T-shaped, π-alkyl and HB	
		TYR-221	HB and Carbon HB	
		ASP-72	π-anion	
		TYR-70	π-sulphur	
		TYR-334	π-π stacked and π-anion	
		TRP-279	π-alkyl	
		SER-286	Halogen (fluorine)	
	BuChE	GLU-238	HB	−11.97
		ASN-245	HB	
		ASN-241	HB	
		PRO-281	π-alkyl	
		VAL-229	π-alkyl	
		PRO-359	π-alkyl	
		TYR-282	π-π stacked and halogen (fluorine)	
		GLY-283	halogen (fluorine)	
Analogue-**14**	AChE	TYR-151	π-sigma	−11.17
		HIS-305	HB	
		ALA-198	π-alkyl	
		LYS-200	π-alkyl	
		ILE-235	π-sigma	
		LEU-162	π-alkyl	
		TRP-59	π-alkyl	
		HIS-201	π-cation	
	BuChE	TRP-329	π-alkyl	−10.87
		PHE-601	π-alkyl	
		ASP-568	π-cation	
		TRP-432	π-sigma	
		ASP-568	Carbon HB	
		ASP-232	π-cation	
		ALA-231	π-alkyl	
		SER-505	Carbon HB	
		ILE-233	π-sigma and π-π T shaped	
		LYS-506	π-anion and π-π T-shaped	
		LYS-506	π-alkyl	
		PHE-476	π-π T-shaped and π-alkyl	
Analogue-**6**	AChE	TYR-151	π-sigma	−10.12
		GLY-306	Carbon HB	
		HIS-305	HB	
		ALA-198	π-alkyl	
		LYS-200	π-alkyl	
		ILE-235	π-sigma	
		HIS-201	π-cation	
		LEU-162	π-alkyl	
		TRP-59	π-alkyl	
	BuChE	VAL-501	π-alkyl	−9.76
		ILE-233	π-alkyl	
		LYS-506	π-alkyl	
		TRP-329	π-π T-shaped	
		PHE-601	π-π T-shaped	
		ASP-568	π-anion	
		ASP-568	HB	
		PHE-476	π-π stacked and π-alkyl	

## Supplementary Materials

The following supporting information can be downloaded at: https://www.mdpi.com/article/10.3390/molecules28207015/s1. Figure S1: 1HNMR for the compound **2**; Figure S2: 13CNMR for the compound **4**; Figure S3: 1HNMR for the compound **7**; Figure S4: 13CNMR for the compound **11**; Figure S5: 13CNMR for the compound **12**; Figure S6: 1HNMR for the compound **16**.
