# Supplementary material for: Design, Synthesis, In Vitro Biological Evaluation and In Silico Molecular Docking Study of Benzimidazole-Based Oxazole Analogues: A Promising Acetylcholinesterase and Butyrylcholinesterase Inhibitors
Source: Molecules. 2023 Oct 10;28(20):7015. doi: 10.3390/molecules28207015 (PMC10609608; doi:10.3390/molecules28207015)
Supplement: Supplementary file 1 [file molecules-28-07015-s001.zip › molecules-2578262-supplementary.pdf]

Design, synthesis, *in vitro* biological evaluation and *in silico* molecular docking study of benzimidazole based oxazole analogues: A promising acetylcholinesterase and butyrylcholinesterase inhibitors

Supplementary Information

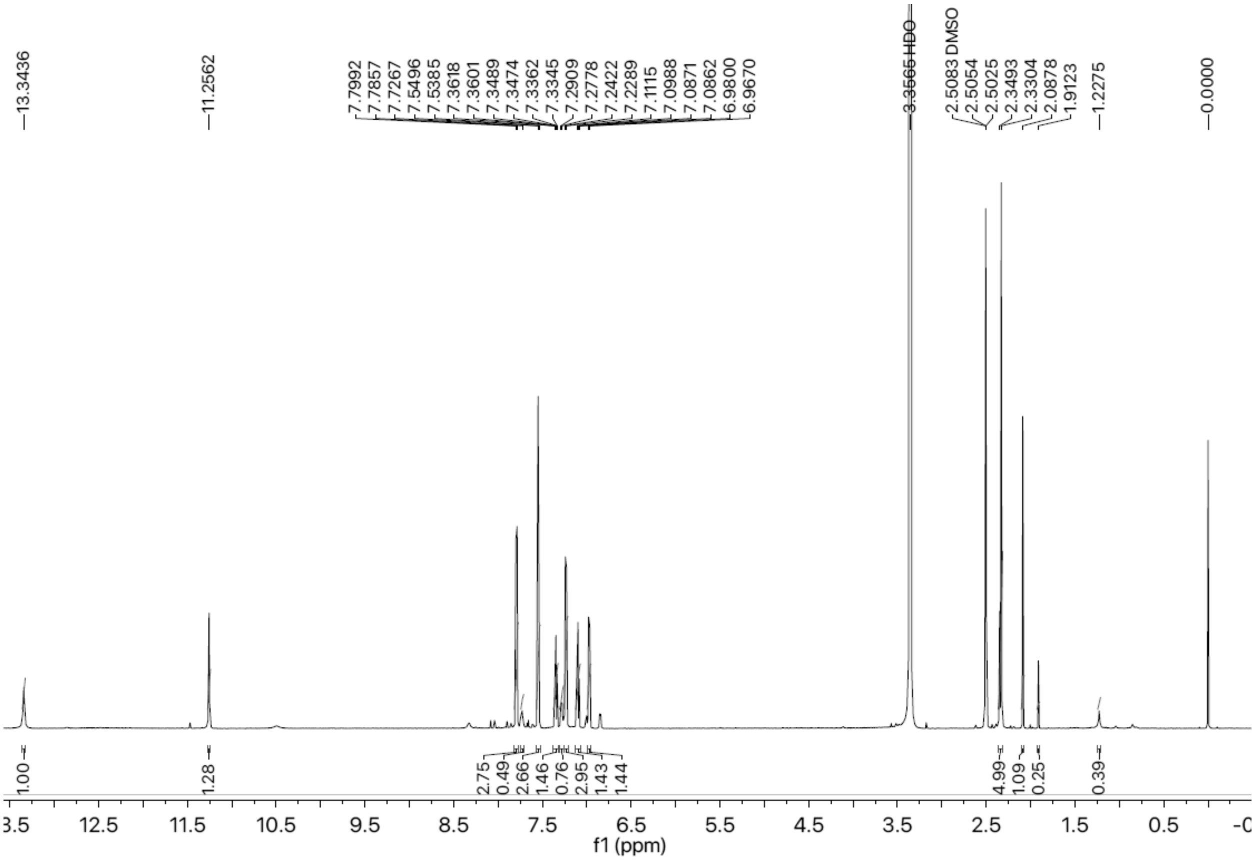

Figure S1. 1HNMR for the compound **2** *(E)-2-(2-(2-((1H-benzo[d]imidazol-2-yl)thio)-1-(p-tolyl)ethylidene) hydrazinyl)-4-(2-nitrophenyl)oxazole* .

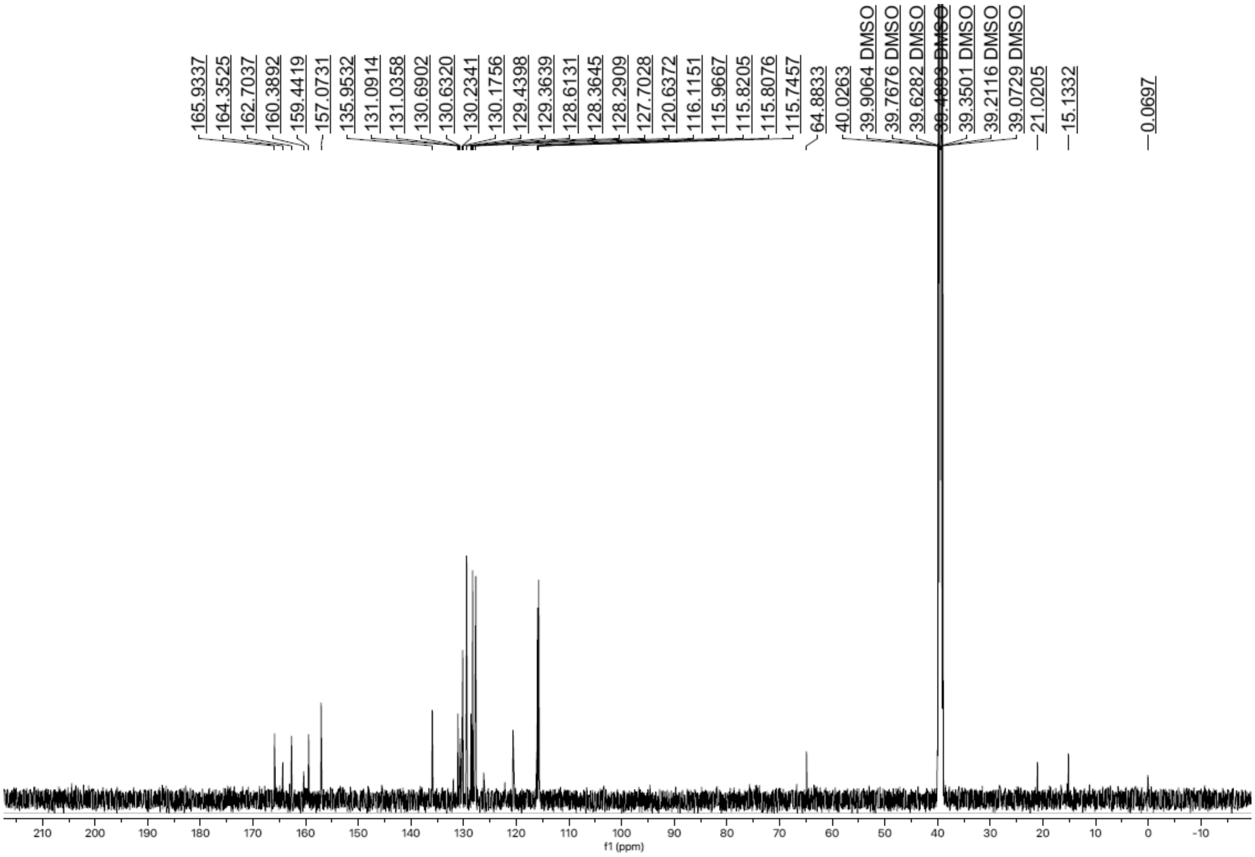

Figure S2. ^{13}CNMR for the compound **4** *(E)-2-(2-(2-((1H-benzo[d]imidazol-2-yl)thio)-1-(p-tolyl)ethylidene)hydrazinyl)-4-(p-tolyl)oxazole* .

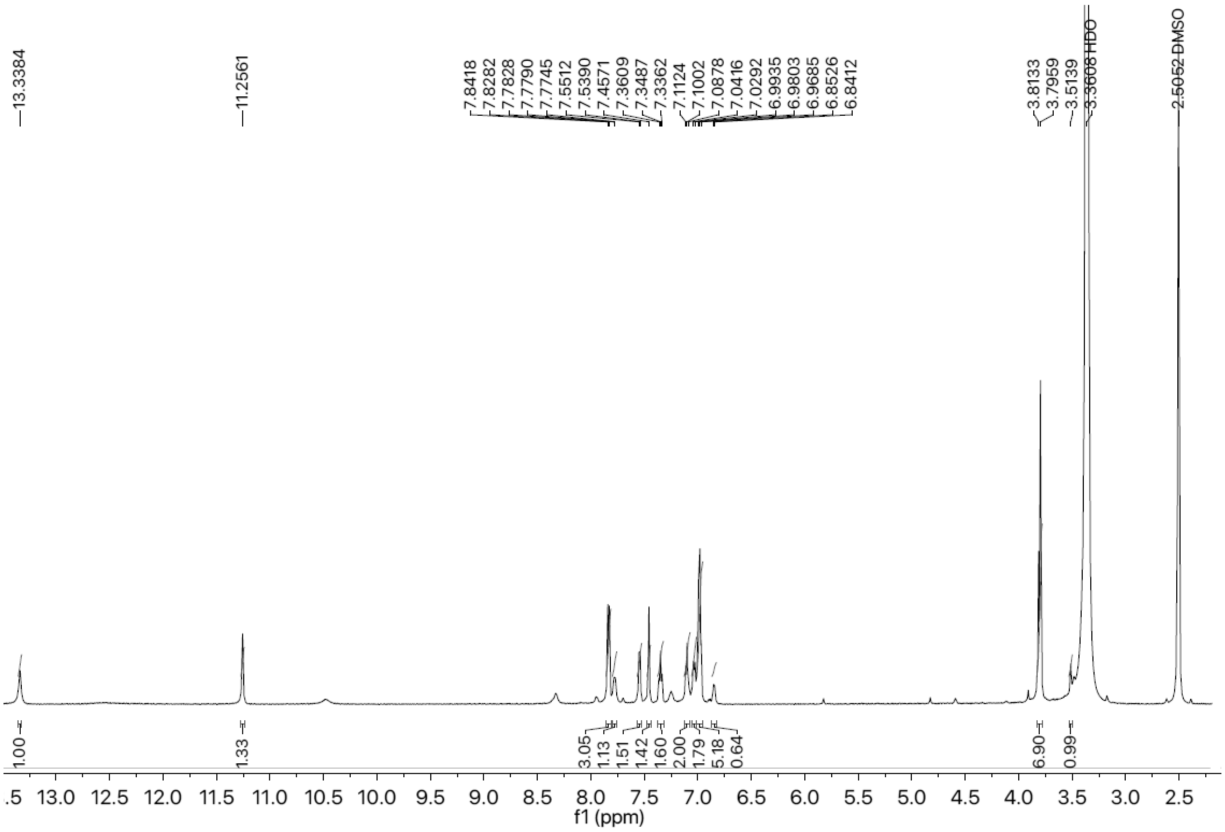

Figure S3. 1HNMR for the compound **7** *(E)-4-(2-((1H-benzo[d]imidazol-2-yl)thio)-1-(2-(4-(4-bromophenyl)oxazol-2-yl)hydrazono)ethyl)-N,N-dimethylaniline* .

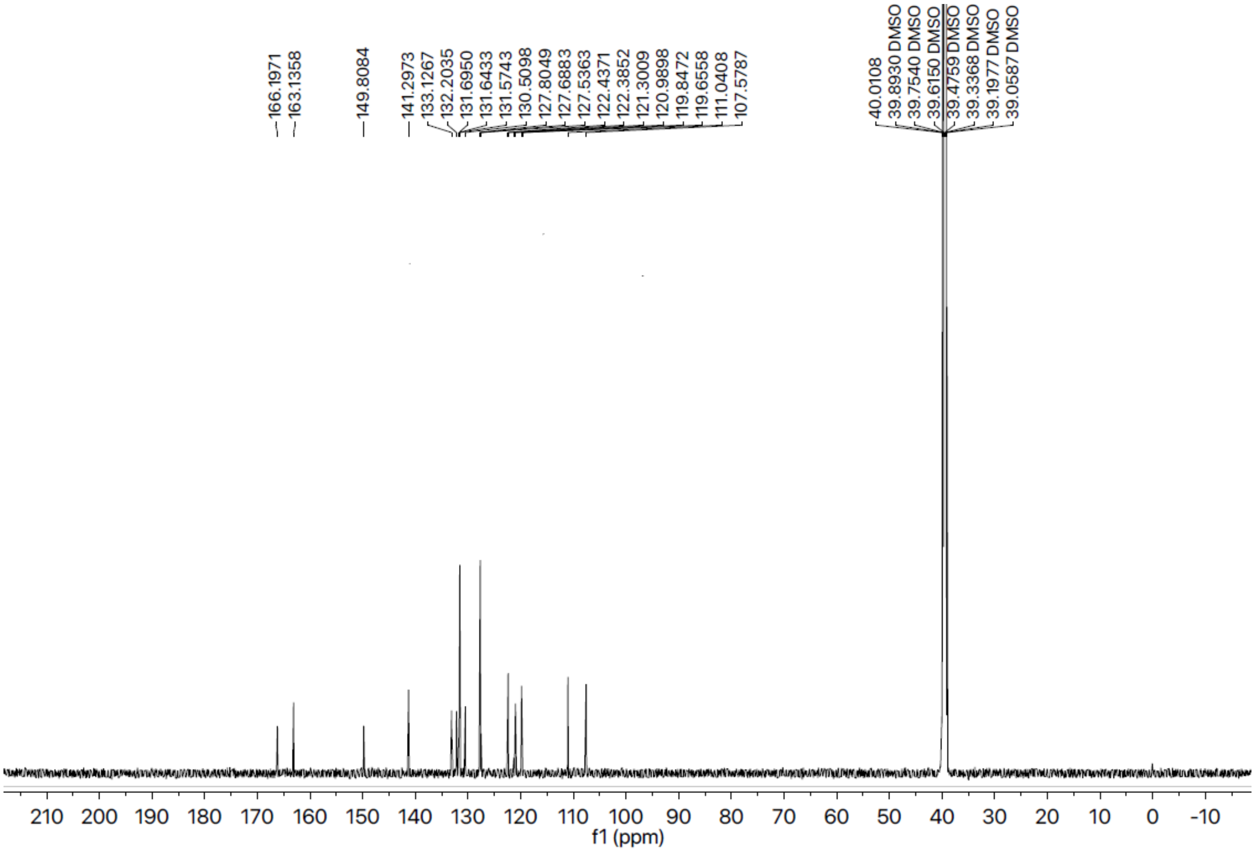

Figure S4. ^{13}CNMR for the compound **11** *(E)-2-(2-(2-((1H-benzo[d]imidazol-2-yl)thio)-1-(4-chlorophenyl)ethylidene) hydrazinyl)-4-(3-nitrophenyl)oxazole* .

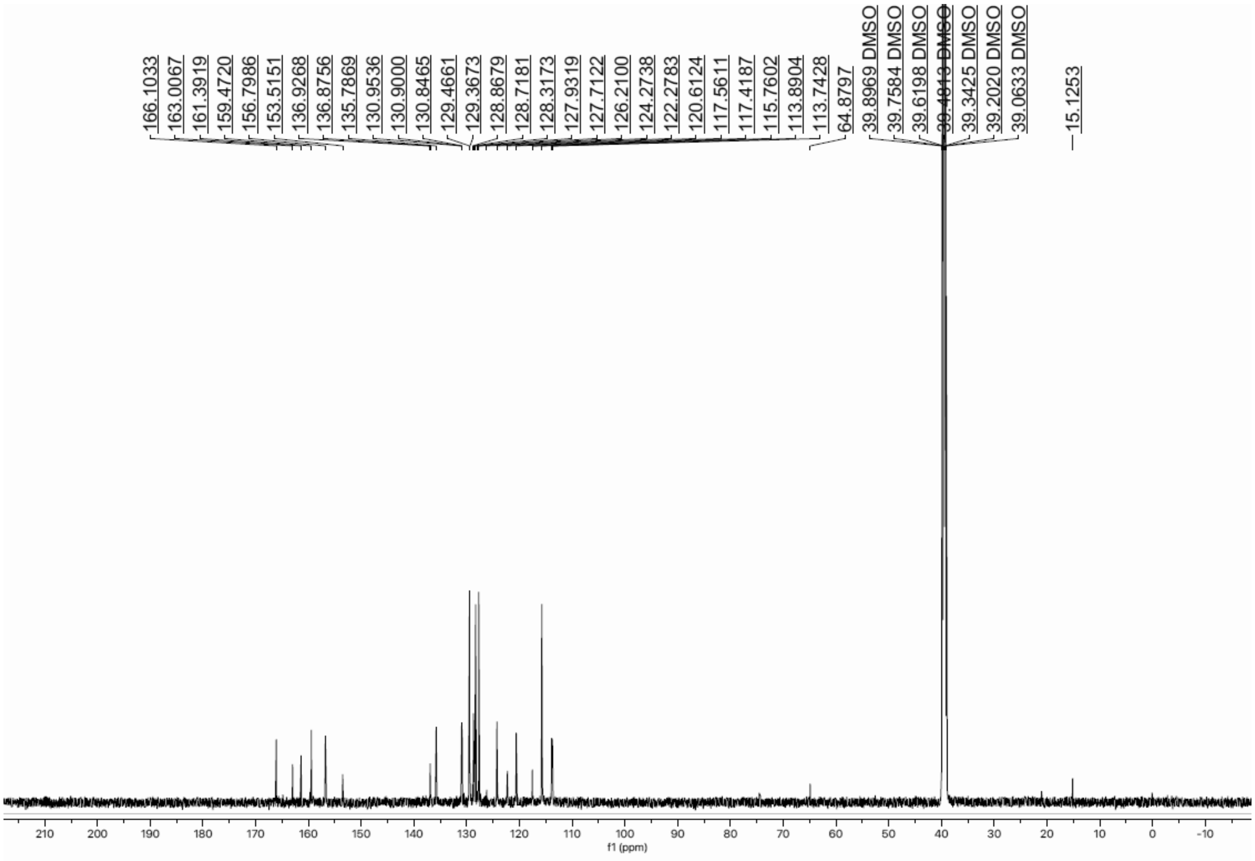

Figure S5. ^{13}CNMR for the compound **12** *(E)-2-(2-(2-((1H-benzo[d]imidazol-2-yl)thio)-1-(p-tolyl)ethylidene)hydrazinyl)-4-(4-chlorophenyl)oxazole* .

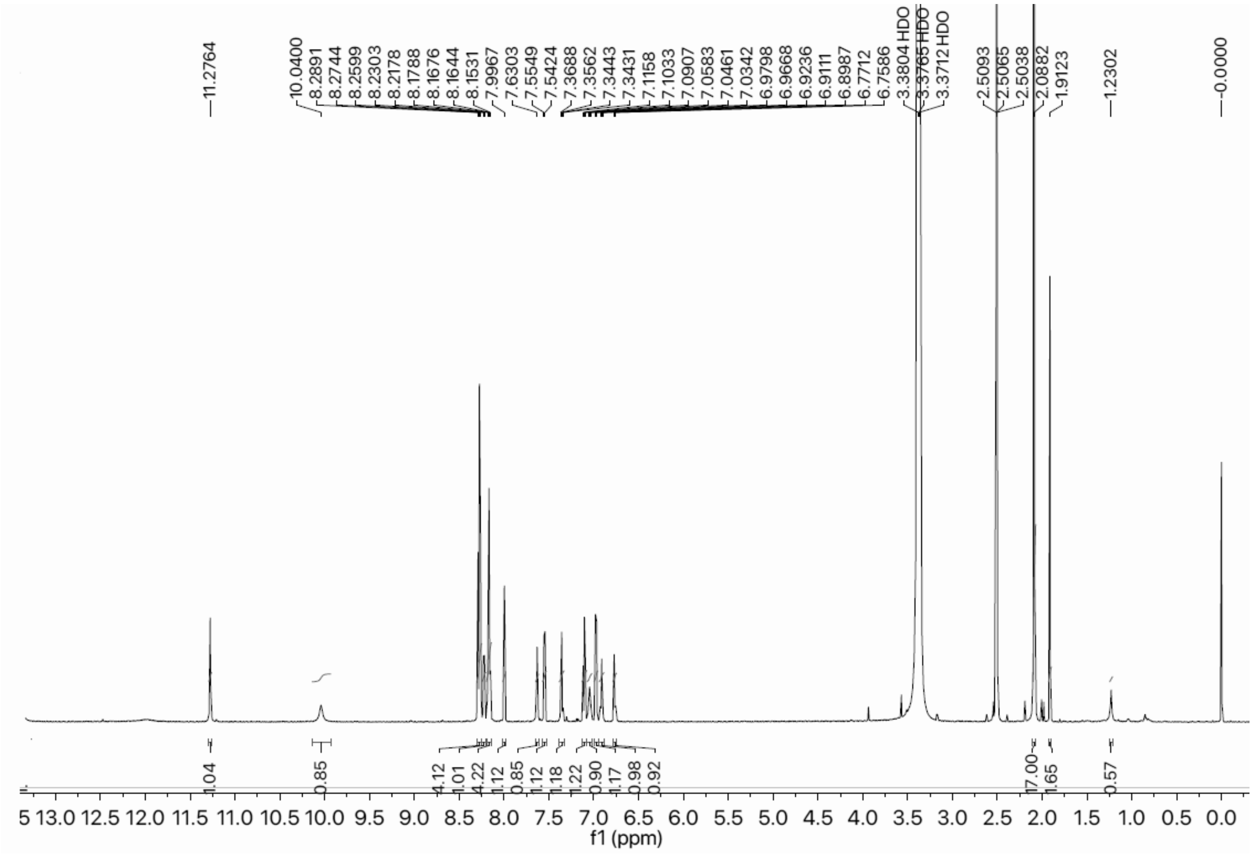

Figure S6. 1HNMR for the compound 16.
